# Structure and Gating Dynamics of Na^+^/Cl^–^ Coupled Neurotransmitter Transporters

**DOI:** 10.3389/fmolb.2019.00080

**Published:** 2019-09-06

**Authors:** Deepthi Joseph, Shabareesh Pidathala, Aditya Kumar Mallela, Aravind Penmatsa

**Affiliations:** Molecular Biophysics Unit, Indian Institute of Science, Bangalore, India

**Keywords:** solute carrier 6 (SLC6), neurotransmitter sodium symporters (NSSs), alternating-access, antidepressants, psychostimulants, secondary active transport

## Abstract

Neurotransmitters released at the neural synapse through vesicle exocytosis are spatiotemporally controlled by the action of neurotransmitter transporters. Integral membrane proteins of the solute carrier 6 (SLC6) family are involved in the sodium and chloride coupled uptake of biogenic amine neurotransmitters including dopamine, serotonin, noradrenaline and inhibitory neurotransmitters including glycine and γ-amino butyric acid. This ion-coupled symport works through a well-orchestrated gating of substrate through alternating-access, which is mediated through movements of helices that resemble a rocking-bundle. A large array of commercially prescribed drugs and psychostimulants selectively target neurotransmitter transporters thereby modulating their levels in the synaptic space. Drug-induced changes in the synaptic neurotransmitter levels can be used to treat depression or neuropathic pain whereas in some instances prolonged usage can lead to habituation. Earlier structural studies of bacterial neurotransmitter transporter homolog LeuT and recent structure elucidation of the *Drosophila* dopamine transporter (dDAT) and human serotonin transporter (hSERT) have yielded a wealth of information in understanding the transport and inhibition mechanism of neurotransmitter transporters. Computational studies based on the structures of dDAT and hSERT have shed light on the dynamics of varied components of these molecular gates in affecting the uphill transport of neurotransmitters. This review seeks to address structural dynamics of neurotransmitter transporters at the extracellular and intracellular gates and the effect of inhibitors on the ligand-binding pocket. We also delve into the effect of additional factors including lipids and cytosolic domains that influence the translocation of neurotransmitters across the membrane.

## Introduction

**Figure 1:**
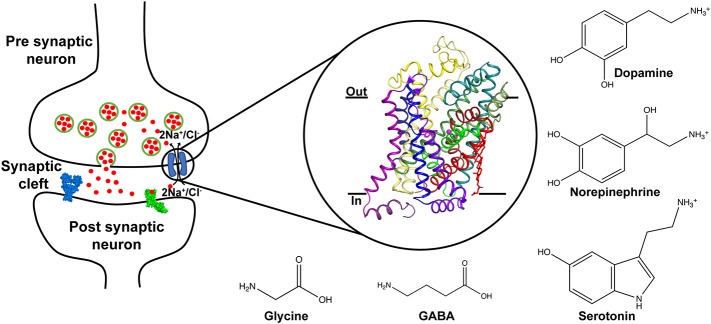
Schematic displaying the role of neurotransmitter transporters in the neural synapse where they control the levels of released neurotransmitters through vesicle exocytosis. The transport is coupled to sodium and chloride gradients. Inset shows the X-ray structure of the dDAT comprising 12 TM helices and bound to dopamine (PDB ID: 4XP1). Chemical structures of neurotransmitters transported through the SLC6 family are depicted.

Chemical neurotransmission between neurons involves the exocytic release of neurotransmitters that activate downstream ligand-gated ion channels and metabotropic receptors (Jessell and Kandel, 1993). The levels of released neurotransmitters are dynamically regulated by the activity of ion-coupled neurotransmitter transporters that couple the Na^+^/Cl^−^ electrochemical gradients to drive the uphill transport of excitatory and inhibitory neurotransmitters (Masson et al., 1999) (Figure 1). A majority of neurotransmitter transporters belong to the SLC6 family (Broer and Gether, 2012) that includes dopamine (DA), norepinephrine (NE), serotonin (5HT), glycine and γ-amino butyric acid (GABA) transporters (Figure 1). The monoamine neurotransmitters play vital roles in maintaining normal locomotor function, sleep cycle, mood, incentive-driven learning and reward (Hornykiewicz, 1966; Jacobs and Azmitia, 1992; Torres et al., 2003). Compromised levels of neurotransmitters cause shifts in neurophysiology that manifest as disorders including major depressive disorder, epilepsy, Parkinson's disease, attention deficit hyperactivity disorder (ADHD) and neuropathic pain (Iversen, 2000, 2006). For instance, reduced monoamine levels are a well-established, albeit not the only source of depression, popularly referred to as the “monoamine hypothesis” (Schildkraut, 1965). Drugs that treat these conditions are inhibitors of neurotransmitter transporters and lead to enhanced levels of monoamines in the neural synapse (Torres et al., 2003).

Incidentally, this is also the mechanism through which psychostimulants act on the dopaminergic pathways of the brain leading to prolonged activation of reward pathways leading to habit formation (Giros and Caron, 1993; Nutt et al., 2015).

**Figure 2:**
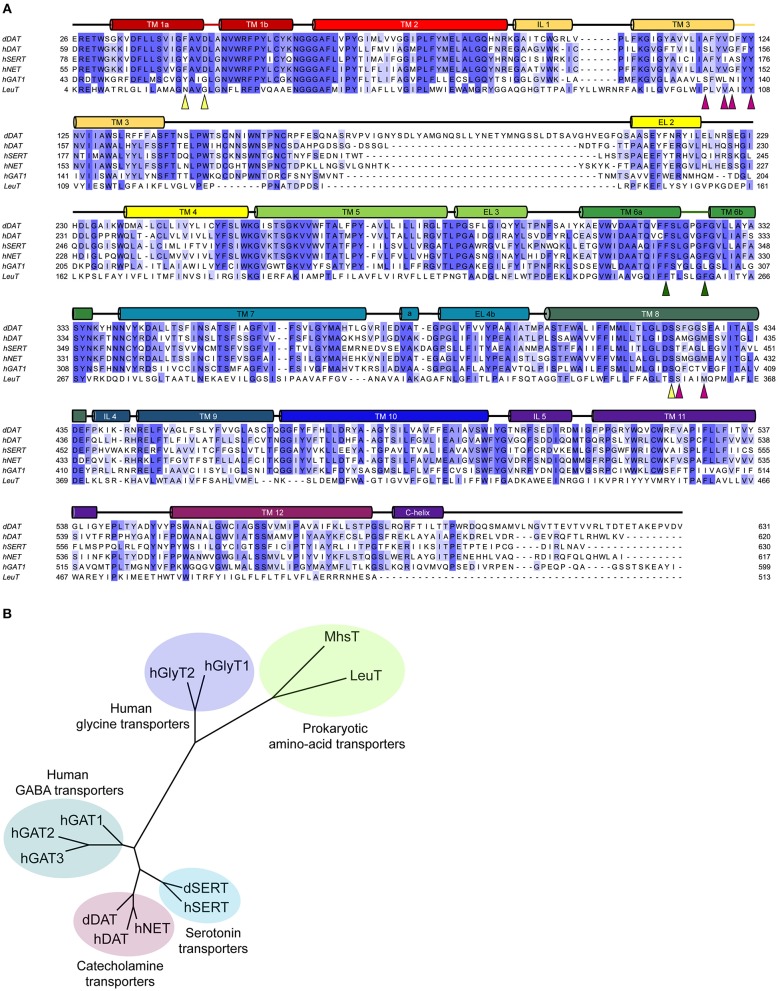
**(A)** Multiple sequence alignment of the eukaryotic NSS members with their prokaryotic homolog, LeuT. The colored cylinders represent the transmembrane regions in accordance to the dDAT crystal structure, colored lines represent discontinuous regions within helices and black lines represent the loop regions. Colored triangles highlight the residues involved in the substrate binding subsites A (yellow), B (magenta), and C (green). **(B)** Phylogenetic analysis of the eukaryotic and prokaryotic NSS members.

**Figure 3:**
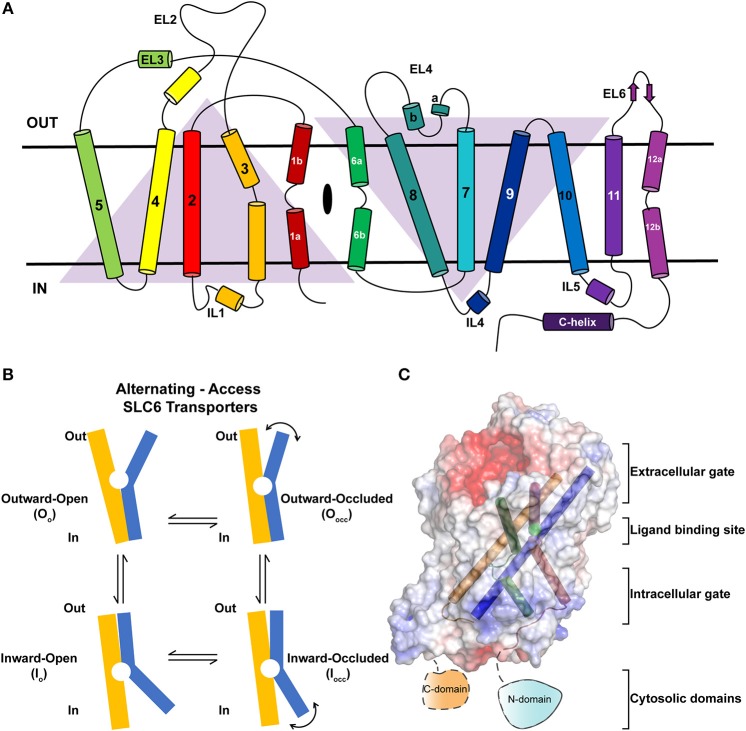
**(A)** Topology of neurotransmitter transporters. Discontinuous helices TM1 and TM6 are involved in neurotransmitter recognition. TMs 3, 8, and 10 serve as scaffold helices in the structure. TMs 1–5 and 6–10 display a pseudo-2-fold symmetry. **(B)** Schematic showing the rocking-bundle type of alternating-access mechanism employed by the NSS members for the transport of substrates. The transport cycle samples multiple conformational states including O_o_, O_occ_, I_occ_, I_o_ to accomplish transport. **(C)** Surface electrostatics of dDAT with gating and scaffold helices are represented in different colors (PDB ID: 4XP1). The figure summarizes the discrete regions of the structure that undergo dynamic movements to elicit substrate translocation.

Neurotransmitter sodium symporters (NSSs) are members of the solute carrier 6 (SLC6) family transporters that comprise around 600–800 amino acids in their primary sequence. The TM regions display significant sequence identities (~50–70%) even amongst NSSs that transport diverse substrates (Figures 2A,B) (Kristensen et al., 2011). Most of the variations betweensequences lie in the unique termini and extracellular loop regions of the transporters (Figure 2A). NSSs are related to prokaryotic amino acid transporters like LeuT with a sequence identity of ~20% (Yamashita et al., 2005; Penmatsa et al., 2013). Within eukaryotic NSS members, sequence identities extend between 50–70% among transporters and the molecular architecture resembles LeuT, a bacterial homolog involved in sodium-coupled amino acid symport (Kristensen et al., 2011). The structural and mechanistic similarities with LeuT have typified the underlying mechanisms of substrate transport in eukaryotic neurotransmitter transporters (Focke et al., 2013; Penmatsa and Gouaux, 2014; Navratna and Gouaux, 2019). The architecture of Na^+^/Cl^−^ coupled neurotransmitter transporters reveal 12 TM helices of which helical bundles 1–5 and 6–10 display a pseudo 2-fold symmetry that allows, “alternating-access” within this family of transporters (Figure 3A) (Jardetzky, 1966; Drew and Boudker, 2016; Majumder et al., 2018). Within the symmetric helices, TMs 1 and 6 serve as gating helices with a non-helical junction in the center that allows movement of extracellular and intracellular halves independent of each other to regulate the exposure and closure of the ligand-binding pocket to solvent access. These movements allow SLC6 transporters to sample outward-open (O_o_), outward-occluded (O_occ_), Inward-occluded (I_occ_) and Inward-open (I_o_) states to establish the transport cycle (Figure 3B) (Yamashita et al., 2005; Krishnamurthy and Gouaux, 2012; Penmatsa and Gouaux, 2014). Recent structural studies of the *Drosophila melanogaster* dopamine transporter (dDAT) (Penmatsa et al., 2013) and human serotonin transporter (hSERT) (Coleman et al., 2016) in complex with multiple transport blockers and altered conformational states have given remarkable insights into the translocation and inhibitory mechanisms in this family. More specifically, the dDAT and hSERT structures reveal the similarities within the gating properties with LeuT and the dissimilarities in the organization of unique structural motifs including the extracellular loop (EL) 2, TM12, and the C-terminal latch (Penmatsa et al., 2013; Coleman et al., 2016). The elucidation of high-resolution crystal structures of the two monoamine transporters facilitated extensive computational and experimental studies into the dynamics that drive substrate transport and inhibitor interactions within the SLC6 family (Grouleff et al., 2015; Cheng and Bahar, 2019). Substrate gating occurs both at the extracellular and intracellular gates that exhibit a propensity to move in or out to proportionately reduce or enhance solvent access to substrate on either side of the neural membrane.

In this review, we highlight the components within the SLC6 monoamine transporters that serve as gates for biogenic amines to move from the synaptic space into the cytosol of neurons and surrounding glial cells. The review seeks to provide a detailed insight into the structure and dynamics of the extracellular gates, ligand binding site, intracellular gates and intracellular domains of monoamine neurotransmitter transporters and extraneous factors that influence their dynamics and role in substrate translocation (Figure 3C). At each of the sections, the review draws comparison between LeuT, dDAT, and hSERT crystal structures to highlight both the parallels and differences involved in their transport properties.

## NSS Architecture

Structural similarities between LeuT and eukaryotic NSSs are particularly prominent in the organization of symmetry related helices 1–5 and 6–10 as they share a similar topology (Figure 3A). A large extracellular loop (EL2) is observed between TMs 3 and 4, which is heavily N-glycosylated in dDAT and other vertebrate NSS members (Melikian et al., 1996; Porzgen et al., 2001). Most structural studies employ trimming this region to a stretch that allows retention of a functional transporter with the shortest possible region of EL2 (Penmatsa et al., 2013; Coleman et al., 2016). The loop decorates the extracellular face of the transporter and closely interacts with EL6 and EL4. Disruption of these interactions by excessive shortening of the loop, as observed in dDAT, leads to a loss of transport activity (Wang et al., 2015). Presence of a disulfide is a common feature in the EL2 of NSS members. In the case of mammalian DATs, the EL2 (H193, D206) forms a Zn^2+^ binding site with EL4 (H375, E396) (Norregaard et al., 1998; Stockner et al., 2013). Binding of Zn^2+^ to this site is observed to inhibit substrate translocation, suggesting a metal ion driven regulation of transport activity (Norregaard et al., 1998). The Zn^2+^ binding site is unique to mammalian DATs and is not observed in other NSS members.

**Figure 4:**
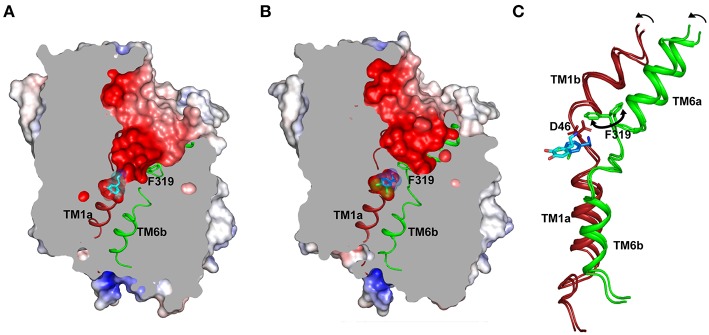
**(A)** Structure displays the outward-open conformation of dopamine bound dDAT with the primary substrate binding site open to the extracellular vestibule and solvent access. The gating residue F319 (TM6, green) is displayed to indicate its role in control of the extracellular gates (PDB ID: 4XP1). **(B)** Inward movement of F319 occludes the binding pocket and separates the substrate analog (DCP) from extracellular vestibule (PDB ID: 4XPA). **(C)** Inward movement in the gating helices TM1b, 6a to form an occluded state. F319 swings in (100°) to occlude the binding site. The D46 undergoes a 120° torsional angle shift to interact with the primary amine of dopamine.

In particular, the gating helices TM1a, 1b and TM6a, 6b that together form the “rocking-bundle,” have a substantial similarity in their organization amongst LeuT, dDAT and hSERT (Forrest et al., 2008). The concerted inward movement of TM1b and TM6a affects the solvent-accessibility of the extracellular vestibule that allows the transporter to transit from an outward open (O_o_) to an outward occluded state (O_occ_) (Figure 3B). While this inward or outward movement of helices occurs by 5.6–7° in dDAT (Figure 4C), 5.5–9° in LeuT and 3–5° in hSERT, the movement occurs in response to substrate interactions in the primary binding site whilst competitive and non-competitive inhibitors prevent this conformational shift and closure of the extracellular vestibule. The gating helices have a disordered region and resemble a pair of “nunchucks” where co-transported ions bind in addition to the substrates and the ligand is ensconced by scaffolding helices, TMs 3, 8 and 10 around them. The binding site has determinants for substrate recognition and inhibitor binding and is subdivided into subsites A, B and C (Sorensen et al., 2012). Further details of subsites would be discussed in a subsequent section. The substrate-binding site is conjoined to the ion-binding sites that consists of two sodium ions and one chloride ion that are co-transported with the substrate in the transport cycle. Release of the substrate is driven by the opening of the cytosolic gates mediated primarily by TM1a (Krishnamurthy and Gouaux, 2012) and unwinding of TM5 to facilitate this movement (Malinauskaite et al., 2014).

The movement of the cytosolic gate is controlled through an electrostatic network that is propagated at the cytosolic face of the transporter with interactions between TM1, IL4, and C-terminal latch (Penmatsa et al., 2013). The C-terminal latch is an extension of the TM12 that caps the C-terminal face of the transporter through ionic and aromatic interactions in both dDAT and hSERT (Penmatsa et al., 2013; Coleman et al., 2016). The W597 (dDAT) forms NH-π interactions with R101 (dDAT) in the intracellular loop 1 (IL1) that in turn forms an electrostatic interaction with the D25 (dDAT) at the N-terminus. Similarly R27 (dDAT), also at the N-terminus, forms electrostatic interactions with D435 (IL3, dDAT) and K441 (IL3, dDAT). The TM12 in eukaryotic NSSs, unlike LeuT, has a kink in the center that prevents it from having dimeric interactions similar to LeuT (Penmatsa et al., 2013), although recent computational and experimental studies indicate oligomer formation of hSERT in the membrane environment (Laursen et al., 2018; Periole et al., 2018).

## Extracellular Vestibule

### Gating in the Extracellular Vestibule

The extracellular vestibule resembles a funnel lined by helices TM1b, TM6a, TMs 3, 8 and 10 on the periphery and EL4 on the top through close interactions with TM1b and EL6. The vestibule is predominantly negatively charged (Figures 4A,B) to facilitate the movement of the positively charged monoamines and inhibitors into the binding pocket, which is located halfway across the membrane bilayer and remains solvent accessible in the O_o_ state (Figure 4A). The extracellular vestibule is observed to serve as a binding site in a few of the drug-NSS complexes particularly S-citalopram-hSERT complex, tryptophan-LeuT complexes and desvenlafaxine in complex with LeuBAT (LeuT modified to mimic the binding site of biogenic amine transporters) (Wang et al., 2015).

The extracellular gates comprise salt bridges between the gating helix TM1b and scaffold helix TM10 in LeuT and NSS structures (Yamashita et al., 2005; Penmatsa et al., 2013; Coleman et al., 2016). In LeuT, this was observed between R30 and D404. The equivalent interactions in dDAT are not apparent between R52 and D475 in both the dopamine bound O_o_ and DCP bound O_occ_ conformations. However, the salt bridge formation in hSERT is apparent between R104 (TM1b) and E493 (TM10) in the O_o_ and I_o_ structures. This interaction disengages in the case of O_occ_ conformation of hSERT. However, it was observed in simulations with hDAT that salt bridges could form between R85 (TM1b) and D476 (TM10) upon binding to dopamine in the primary substrate binding pocket (Cheng and Bahar, 2015).

The inward movement of the helices TM1b and TM6a in response to substrate interactions induces the coordinated movement of the EL4 region inwards, to partially reduce the solvent access inside the extracellular vestibule. This is more apparent in comparison with the inward open state of LeuT where EL4 moves into the vestibule by nearly 3–4 Å forming close interactions with EL6. The movement of EL4 is closely coupled to the inward movement of TM1b through aromatic stacking interaction between W103 (hSERT) and P403 (hSERT) in the EL4 turn.

Substrate binding in the primary binding site induces closure of the substrate-binding pocket to solvent access leading to an O_occ_ state (Figure 4B). This is predominantly induced by the inward movement of the residue F319 (dDAT) that controls solvent access to the substrate-binding pocket (Figure 4C). Among the available structures of NSS members, LeuT bound to leucine predominantly displays an O_occ_ state and dDAT displays an O_occ_ conformation in the presence of dichlorophenylethylamine (DCP), a halogenated analog of DA. Residue F319 is part of the ligand-binding site and details of this gating process by F319 are discussed as part of the dynamics of the ligand-binding site.

### Allosteric Site

The entrance of the extracellular vestibule is observed in multiple instances in LeuT and hSERT to bind diverse compounds including β-octylglucoside, L-tryptophan, D-maltose, clomipramine and S-citalopram. The clomipramine bound O_occ_ LeuT structure provided insights into antidepressants acting as potential non-competitive inhibitors by blocking the unbinding of the substrate or inhibitor bound in the primary binding site (Singh et al., 2007). Biochemical studies have also suggested the role of high affinity substrate binding to a secondary binding site (S2) in the vestibule that is essential for transport activity. Binding of leucine at this position was proposed as a symport effector that would trigger the release of the substrate bound in the primary binding site (Shi et al., 2008). However, a clear second binding site for the substrate in the vestibule of LeuT was not evident despite extensive crystallographic studies done using LeuT. This was a point of debate in the area with the site being primarily attributed to be non-specific that can bind detergent (Piscitelli et al., 2010). But, proponents of the second binding site suggest it to be playing an important role in the allosteric control of transport properties (Zhao et al., 2011).

**Figure 5:**
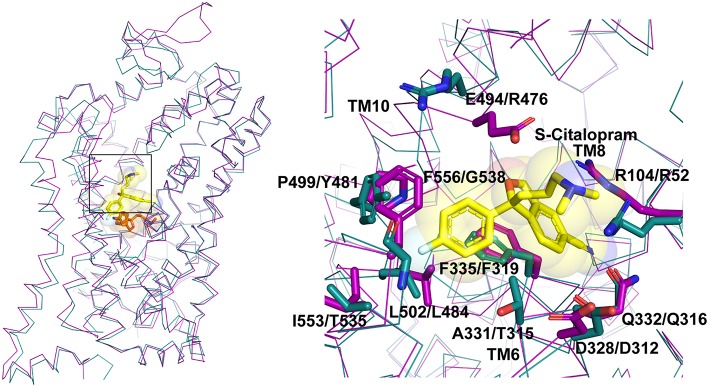
Allosteric binding site comparison between hSERT (magenta) and dDAT (cyan) for the citalopram allosteric site. The residues interacting with S-citalopram are highlighted as sticks in hSERT (PDB ID: 5I73) and their equivalent residues shown in dDAT (PDB ID: 4XP4).

In multiple studies involving simulations, substrates and drugs like cocaine were observed to transiently interact at the S2 site prior to binding to the primary binding site (Cheng et al., 2015). Several biochemical studies hinted at the potential for S-citalopram to bind at an allosteric site and inhibit hSERT activity (Chen et al., 2005; Matthaus et al., 2016). This was conclusively proven in the crystal structure of hSERT in complex with S-citalopram (Coleman et al., 2016), wherein a clear density for the drug was observed in the vestibule coordinated by residues from TMs 1b, 6a, 10 and 11 (Figure 5). Allosteric inhibitors prevent the binding or unbinding of fresh substrate by blocking vestibular entry to the primary binding site, thereby preventing further conformational transitions in the transporter. In S-citalopram, the fluorophenyl group is stacked in a hydrophobic pocket created by TM10 and 11 amidst residues F556, L502, P499 and I553 (Figure 5). The dimethyl propylamine group is observed to face the entrance of the vestibule and is accessible to solvent. While the hSERT crystal structure does not indicate a direct interaction, the tertiary amine could have interactions with E494 in the vicinity. The larger benzofurancarbonitrile group of the drug is stacked in between R104 (TM1b) and F335 (TM6a) that serve as the primary gating residues in the vestibule. R104 forms a salt bridge with E493 and F335 is known to undergo movements to open or close the primary substrate-binding site to solvent access through the extracellular vestibule to form the occluded state. S-citalopram was also observed to stabilize TM1b region in HDX mass spectroscopy, likely due to its ability to interact at the allosteric site, in comparison with cocaine, which only binds in the primary binding site (Wang et al., 2015; Moller et al., 2019). It was also observed that bivalent substrates containing two covalently linked dopamine or serotonin units had significantly higher affinities toward hDAT and hSERT suggesting that the vestibule could harbor an additional interaction site in mammalian NSSs (Andersen et al., 2016). However, X-ray structures of dDAT or other drug complexes with hSERT do not reveal substrate binding to the extracellular vestibule suggesting that this phenomenon might be specific to citalopram-SERT interactions. In fact, the binding site comparisons between dDAT and hSERT at the allosteric site reveal differences in the interactions between EL6, TM11 and TM10 that could explain the absence of a secondary antidepressant binding site in dDAT (Figure 4A). In the absence of drug bound in the allosteric site, a molecule of D-maltose was observed suggesting the potential non-specific interactions in this region of the vestibule. At higher ligand concentrations (20 mM) an allosterically bound desvenlafaxine was observed in LeuBAT (Wang et al., 2013), a LeuT construct with residues engineered to resemble a biogenic amine transporter. However, biochemical analysis of this SNRI clearly reveals the competitive nature of its inhibition (Deecher et al., 2006).

Recent studies have also identified that adenine nucleoside derivatives that are traditionally A3 adenosine receptor agonists can interact specifically with hDAT and hNET to cause transport inhibition, albeit not as competitive inhibitors at the primary binding site. These drugs presumably interact with hDAT in the vestibule that allows increased affinity for competitive inhibitors of DAT like RTI-55 and mazindol (Janowsky et al., 2016; Navratna et al., 2018).

## Substrate or Inhibitor Binding Site

**Figure 6:**
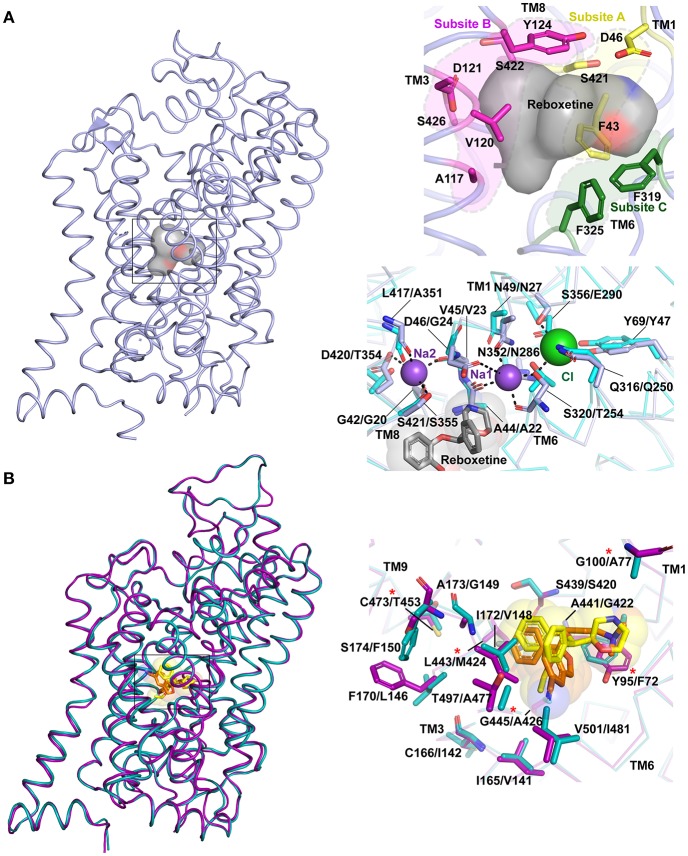
**(A)** Primary binding site in neurotransmitter transporters are subdivided into subsites A (yellow), B (magenta) and C (green). The surface of the bound drug molecule is displayed in gray. Residues involved in the ion binding sites are compared within dDAT (light blue) bound to reboxetine, represented by gray sphere (PDB ID: 4XNX) and LeuT (cyan) (PDB ID: 3F3A). The first residue in description represents dDAT followed by its equivalent in LeuT. **(B)** Differences in binding pockets of hSERT (magenta) bound to S-citalopram (orange) (PDB ID: 5I73) overlapped with dDAT bound to reboxetine (yellow) (PDB ID: 4XNX) and hNET homology model (cyan). The first residue in description represents hSERT followed by its equivalent in hNET. Asterisk represents five hSERT like substitutions in hNET that increase the affinity of hNET toward S-citalopram (Andersen et al., 2011).

The substrate-binding pocket of eukaryotic NSSs is the primary region of interactions for all the monoamine and inhibitory neurotransmitters. Substrate selectivity toward monoamines, glycine and GABA is enforced in this binding site. The funnel like extracellular vestibule opens into a cavernous and flexible binding pocket that is capable of binding not just neurotransmitters, but also antidepressants and psychostimulants (Wang et al., 2013, 2015; Penmatsa et al., 2015). Competitive inhibitors bind with high affinity to the primary substrate binding pocket effectively preventing neurotransmitters from accessing the binding pocket. The steric bulk of the inhibitors also prevent closure of extracellular gates during the transport cycle thereby disallowing formation of the occluded intermediate (Apparsundaram et al., 2008; Penmatsa et al., 2013). In the O_o_ state, the binding pocket is accessible to water molecules, sodium and chloride ions that bind in the immediate vicinity of the substrate or inhibitor. Two sodium ions are bound to the Na1 and Na2 sites in all three experimental structures of LeuT, dDAT and hSERT. Na1 site is ensconced between TM1, 6 and 7 and is coordinated in part by the main chain carbonyl groups of S320, A44 and side chains of S320 (TM6), N49 (TM1), N352 (TM7) in dDAT. The Na2 site is coordinated by residues from TM1 and TM8 through main chain carbonyl groups of D46 (TM1a), F43 (TM1a), L417 (TM8) and side chain of S421 (TM8) and D420 (TM8) in dDAT. In the eukaryotic NSS members an additional chloride ion is bound in the vicinity of Na1 site. The Cl^−^ is held through tetrahedral coordination between Y69 (TM2), Q316 (TM6), S320 (TM6), and S356 (TM7) in dDAT. In LeuT, the negatively charged chloride is substituted through E290 in TM7 (Kantcheva et al., 2013) (Figure 6A). It was observed in LeuT that binding of sodium ions could induce O_o_ state prepped for binding to a substrate molecule. These observations are also confirmed through DEER measurements where substrate free LeuT bound to Na^+^ stays in an outward-open conformation (Claxton et al., 2010). Simulations with hDAT have revealed the existence of a channel-like state in the transporter particularly in the substrate free form where the closure of extracellular gates and intracellular gates is not tight in comparison to substrate bound form (Cheng et al., 2018).

Substrate binding induces an occluded conformation in the transporter, which involves closure of the extracellular gates. The substrate-binding pocket is further divided into three subsites A, B and C based on the regions of inhibitor-protein interactions (Figure 6A) (Wang et al., 2013). Subsite A of dDAT includes D46 (TM1), F43 (TM1), and S421 (TM8) at the bottom of the binding site. Subsite B includes TM3 and TM8 region where a bulk of the hydrophobic moieties wedge. Subsite C comprises of unwound helical region stretching from F319 to F325 in TM6. The division of the primary binding sites into subsites facilitates lucid interpretation of the drug-induced shifts within the binding site. It was also observed that selectivity of inhibitors toward different monoamine transporters is encoded by the residues surrounding the binding pocket. For instance, the K_i_ of S-citalopram for hNET could be substantially improved to match that of WT hSERT by altering five non-conserved residues to their hSERT equivalent residues within the binding pocket of hNET (Figure 6B). NET specific inhibitors like nisoxetine and atomoxetine exhibit a corresponding loss of affinity in this mutated construct (Andersen et al., 2011). Also NET selective inhibitors have a higher affinity to hNET over hDAT due to specific residues in the binding site including A145 and A426 that allow the moieties in nisoxetine and reboxetine to bind; where in hDAT the two residues are serines that disallow the NRIs from binding (Penmatsa et al., 2015).

At this juncture, dDAT structures in complex with dopamine, dopamine analog 3,4 dichlorophenylethyl amine (DCP) and D-amphetamine are the only structures available for interpretation of substrate-induced changes within the binding sites (Figures 4A–C). The hSERT structures are predominantly inhibitor bound outward-open or ibogaine bound inward-open structures. The recognition of the positively charged amine group occurs at the negatively charged D46 (dDAT) (D98 in hSERT) of subsite A. This Asp side chain along with the main chain carbonyl of the F319 (F335 in hSERT) form a local negatively charged area that strongly attracts monoamines and the secondary and tertiary amines of antidepressants and psychostimulants. The binding of dopamine is observed to induce a χ1 torsional angle shift of nearly 120° in D46 of dDAT (Figure 4C). In this process, the aspartate disengages from its coordination with the Na1 site and coordinates with amine group of dopamine. The equivalent residue in case of other NSS members, whose substrates are amino acid neurotransmitters like glycine and GABA, have a substitution at the aspartate residue to glycine to better accommodate the carboxyl group of the amino acid, akin to LeuT. The disordered stretch of TM1 continues to form the floor of the binding pocket F43, which in case of the hSERT is a tyrosine (Y95) that undergoes a massive shift of 6 Å in the Cα position and 12.5 Å in the position of the phenyl group during the conformation change leading to the inward-open (I_o_) state. This movement facilitates solvent access to the binding site and these changes are discussed in detail in the subsequent section dealing with the intracellular gate dynamics.

Subsite B in dDAT is the region of the binding pocket that faces the scaffolding helices TM3 (V120, D121, Y124) and TM8 (S426, S422). The aromatic groups of numerous drugs wedge into this pocket and display strong inhibitory effects on the transport process. Interestingly, subsite B is also the site where some variations are observed between dDAT and other mammalian NSS members. The dDAT subsite B is a relatively polar region that has D121 and S426 residues that interact with the catechol ring of dopamine and presumably noradrenaline. In a multiple sequence alignment, the two residues are a cavity forming, side chain lacking glycine instead of D121 in both hDAT and hNET or a neutral side chain containing alanine (A173) in case of hSERT. S426 is usually a methionine in case of both hDAT and hNET and a leucine (L443) in case of hSERT (Figures 2A, 6B). While the affinity of dDAT is not hampered toward transport inhibitors due to a polar subsite B, the substitution of D121 to glycine or S426 to methionine to mimic hDAT subsite B leads to a significant enhancement of affinity toward drugs like nisoxetine, cocaine and other antidepressants to varying extents (Wang et al., 2015). One of the outcomes of these substitutions is the complete loss of transport activity suggesting its potential role in affecting transport (Wang et al., 2015).

**Figure 7:**
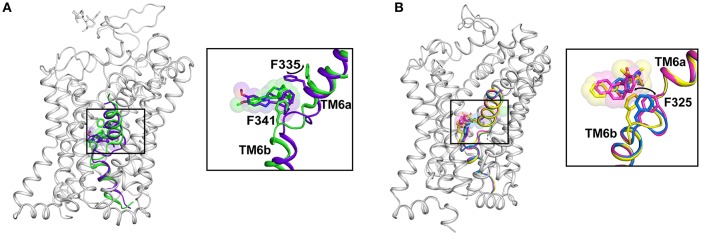
**(A)** Structural changes in the binding site caused by a transition from O_o_ (PDB ID: 6DZY) state to I_o_ (PDB ID: 6DZZ) state in hSERT. The F335 moves in to occlude the binding pocket and lowering of the drug-binding pocket to sense the altered position of ibogaine. **(B)** Plasticity of the binding pocket dictated by movements of subsite C with the F325 sensing the size of the bound ligand through aromatic interactions. Structural differences in the binding pocket with nortriptyline (pink) (PDB ID: 4M48), cocaine (yellow) (PDB ID: 4XP4) and D-amphetamine (blue) (PDB ID: 4XP9).

The subsite C consists of the non-helical junction between TM6a and 6b that exhibits substantial plasticity to accommodate drugs and substrates of varying sizes and affect the conformational changes within the transporter to translocate the neurotransmitter. The residue F319 (dDAT) (F253 in LeuT; F335 in hSERT) serves as a gating residue to occlude the binding pocket from solvent access upon substrate binding. The inward movement of the F319 side chain by about 100°, closes the solvent access of the extracellular vestibule to the substrate-binding pocket (Figure 4). While this movement is consistently observed within LeuT and the substrate analog bound structure of dDAT, the ibogaine bound outward occluded (O_occ_) structure of hSERT does not exhibit any such movement of the side chain, F335. However, the inward open state of hSERT does display this conformational change with the F335 side chain shifting inward to occlude the extracellular vestibule (Figure 7A). The residue that prominently interacts with the drugs through aromatic edge-to-face interactions is F325 (dDAT) and F341 (hSERT). The interactions of this residue with the aromatic groups of the substrate and inhibitor allow the control of the size of the binding pocket, which can accommodate drugs with a small surface area (D-amphetamine, 314.6 Å^2^) to large inhibitors including tricyclic antidepressants (476 Å^2^) (Figure 7B). These movements suggest that the binding site retains plasticity to accommodate substrates and drugs of varying sizes.

## Dynamics of the Intracellular Gates

**Figure 8:**
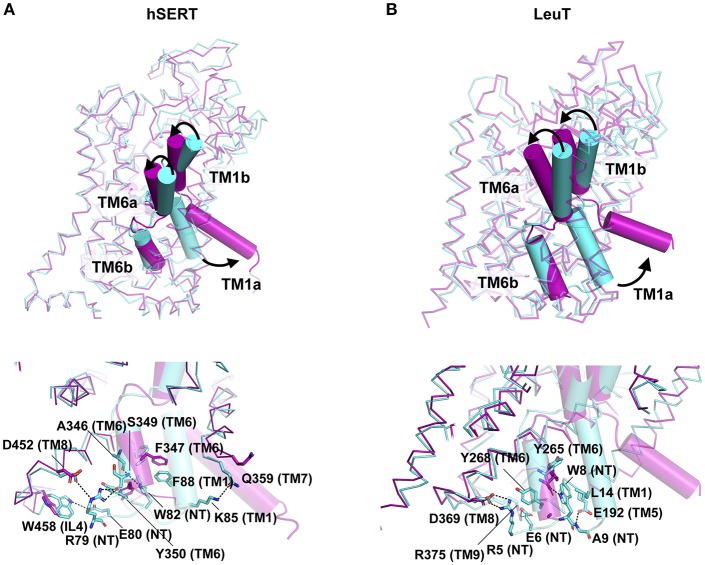
**(A,B)** Extracellular and Cytosolic gating of the substrate in hSERT (PDB ID: 6DZV, 6DZZ) and LeuT (PDB ID: 3TT1, 3TT3) respectively. O_o_ state of the structure is represented in cyan whilst I_o_ state in magenta. Structures display the difference in the conformation as a consequence of TM1b, TM6a closing on the top and TM1a open to release neurotransmitter into the intracellular milieu. Intracellular network of H-bonds and hydrophobic interactions represented in the bottom panel for each molecule.

Unlike the major facilitator superfamily transporters that function as rocking switches to allow vectorial movement of substrate, the LeuT family transporters employ a rocking bundle of two helices (Drew and Boudker, 2016). The transition of NSS transporters from O_occ_ to I_o_ state is triggered by the binding of substrate in the primary binding site of transporters like LeuT and tyrosine transporter Tyt1 (Quick et al., 2006; Zhang et al., 2018). While the inward movement of the TMs 1b and 6a close the extracellular gate, the opening of TM1a facilitates solvent access to the binding site. The X-ray structure determination of the inward open state of LeuT was carried out by adding mutations in the Na2 site (T354V, S355A) and a substitution in the gating residue Y268A followed by the use of conformation specific antibody (Figure 8A) (Krishnamurthy and Gouaux, 2012). The inward open state of the hSERT structure was elucidated in the presence of K^+^ ions and ibogaine, an alkaloid that stabilizes the inward open conformation along with a Fab that interacts with the extracellular face of the transporter to enhance the particle size of the molecule for high resolution cryoEM studies (Coleman et al., 2019). Both LeuT and hSERT structures show the large scale opening of the TM1a after disengaging its salt bridges (R5 in LeuT, R79 in hSERT) with the interaction network. Interactions at the cytoplasmic gate in hSERT involve H-bonds between R79 with main chain carbonyl of S349 (TM6b) and in close proximity to D452 (TM8). The residue E80 forms interactions with indole NH group of W458 (IL4). In hSERT the TM1a residue W82 forms a continuous edge-to-face aromatic stack with F88 (TM1a) and Y350 (TM6b) (Figure 8A). These interactions are lost during the opening of the cytosolic gate that lead to a massive movement resembling a “trapdoor,” that swings the TM1a out by 40° to allow the displacement of the floor of the binding pocket Y95 and providing solvent access to the binding site through the cytosol (Figures 8A,B). The outward movement of the TM1a is apparent in the high-resolution cryoEM study published by Coleman et al. (2019) that provides a remarkable insight into the gating dynamics of neurotransmitter transporters and trap a rather fleeting I_o_ state, in solution (Figure 8A). During the process of opening the intracellular gate, TM6b remains unchanged in its position. The opening of TM1a is associated with the partial unwinding and splaying of the TM5 helix, moving the TM1a outward. In the hSERT I_o_ structure, one can see unwinding of TM5 at the cytosolic face between residues 272 to 283. The TM5 residue W282, in the I_o_ conformation stacks against the TM1a that has swung out to provide solvent access to the substrate-binding pocket. The unwound portion of the helix also partially and sterically compensates for the void created by the movement of TM1a, as observed in the I_o_ structure (Coleman et al., 2019). The unwinding of TM5 was also observed in the case of the I_occ_ state structure of MhsT, a tryptophan transporter from *Bacillus halodurans* (Malinauskaite et al., 2014). The transporter displays an unwinding of the TM5 at the Gly-X9-Pro motif allowing partial solvent access to the Na2 site triggering the dissociation of Na^+^ ion. Unwinding of helices TM7 and TM6a along with TMs 1a and 5 were observed in the HDX-MS studies of LeuT, during its transition from an outward occluded (O_occ_) state to inward open (I_o_) state (Merkle et al., 2018). MD simulations have shown that dissociation of the Na^+^ from site 2 is essential to initiate the opening of the intracellular gates (Watanabe et al., 2010). The interaction network at the intracellular gate is vital for establishing a productive transport cycle. This is evident from the case of a congenital mutation in the dopamine transporter involving a single residue deletion ΔN336 in the IL3 of the transporter that results in a transport defective mutant leading to autism spectrum disorder. This deletion, induces the formation of a previously unobserved half-open inward-facing (HOIF) conformation that is transport inactive (Campbell et al., 2019).

**Figure 9:**
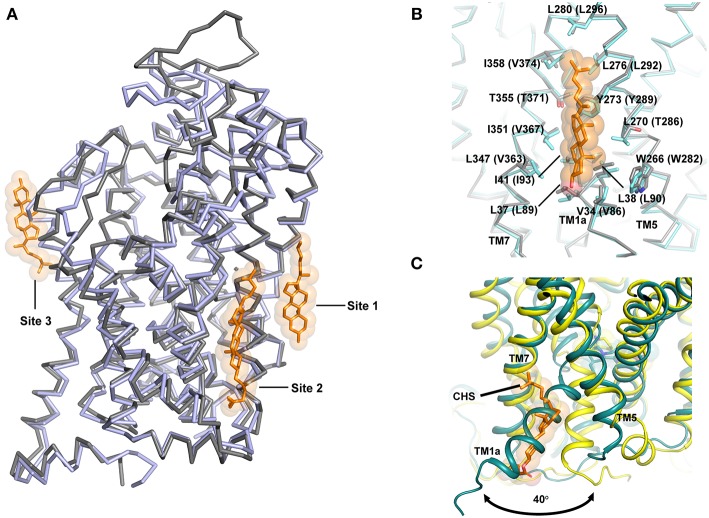
**(A)** Cholesterol interactions observed in the NSS structures hSERT and dDAT. Cholesterol sites 1 and 2 are observed in dDAT (PDB ID: 4XP1) in the inner leaflet while site 3 is observed at TM12 region in hSERT structure (PDB ID: 5I73). **(B)** Site 1 is anchored in the junction between TM1, 7, and 5 and the figure displays similarities in the binding sites of both dDAT and hSERT. **(C)** Movement of the TM1a in hSERT structure is facilitated by absence of cholesterol. The cholesteryl hemisuccinate (CHS) was observed in the O_occ_ structure of hSERT (PDB ID: 6DZV).

Another major factor that influences the ability to open or close is the interaction of TM1a with cholesterol (Figure 9). In fact, the presence of cholesterol in the ibogaine bound structure of hSERT retains it in an outward-open state (Coleman et al., 2019). The availability of the inward open state of the hSERT provides the first glimpse of the complete transport mechanism of eukaryotic neurotransmitter transporters.

## Role of Lipids in Transport Function

It is well known that transport activity and inhibitor binding in neurotransmitter transporters are significantly influenced by membrane cholesterol (Scanlon et al., 2001). It was also demonstrated prior to the structure determination of dDAT that cholesterol stabilizes an outward-open inhibitor bound conformation of the neurotransmitter transporters (Hong and Amara, 2010). One of the most interesting observations from the dDAT structure is the identification of a cholesterol-binding site in the inner leaflet in close proximity with TM1a in the junction formed by TM5 and TM7. The β-face of the sterol ring of cholesterol is lined with residues W266, L270, Y273 from TM5 and V34, L37, L38, I41 from TM1a of dDAT (Figures 9A,B). The isooctyl group of cholesterol is docked at the interface of TMs 5 and 7 by residues L276, L277, and I358 of dDAT (Penmatsa et al., 2013). The extent of conservation between dDAT and hSERT is extensive in cholesterol site 1 (Figure 9A). An additional cholesterol binding site was observed in dDAT structures adjacent to site 1 interacting closely with TMs 2, 7 and 10, referred to as site 2. However, in hSERT O_o_ crystal structures, bound cholesterol was not observed at sites 1 and 2 as seen in dDAT, although an additional cholesterol binding site was observed in the outer leaflet near TM12a, referred to as site 3 (Figure 9A) (Coleman et al., 2016). In the recent hSERT structures (Coleman et al., 2019), elucidated through cryoEM, a cholesterol molecule is observed in the putative occluded state of the transporter bound to ibogaine at site1, further reinforcing this site as a major point of control for the conformational flexibility of the transporter (Figure 7B). Coarse-grained molecular dynamics have identified two additional sites on the transporter surface that are prone to interact with cholesterol (Zeppelin et al., 2018).

Given the understanding of the transport process gained from LeuT in multiple conformations, it is evident that TM1a is the primary motif that swings out by 45° to create solvent accessibility to the substrate binding pocket. In the presence of cholesterol at site 1, this outward movement of TM1a would be sterically blocked (Figure 9C). In the recent past, this premise was tested by *in silico* and biochemical studies wherein mutations in the cholesterol-binding site 1 resulted in the enrichment of the inward-facing conformation (Laursen et al., 2018; Zeppelin et al., 2018). MD simulations with hDAT model also show that cholesterol site 1 is the most likely binding site for cholesterol in monoamine transporters (Zeppelin et al., 2018; Schumann-Gillett et al., 2019). Simulations performed in the absence of cholesterol lead to the early formation of an I_o_ conformation with unwinding of the TM5 cytosolic region leading to solvent accessibility at the Na2 site (Malinauskaite et al., 2014; Zeppelin et al., 2018).

In addition to cholesterol, a few other studies have shown the ability and importance of PIP2 in mediating some aspects of hDAT function, particularly its ability to display amphetamine induced dopamine efflux and in oligomerization. PIP2 is suggested to interact with the hDAT N-terminus that has substantial numbers of positively charged residues for the anionic PIP2 head group to interact with (Khelashvili et al., 2015). Computational analyses identified that this interaction can stabilize additional intermediates in the ion-release pathway thereby facilitating altered Na^+^ release pathways leading to dopamine transport (Razavi et al., 2018). Also, PIP2 interactions are suggested to mediate stable oligomer formation with hSERT (Anderluh et al., 2017) although hDAT oligomerization is observed to be PIP2 independent in single-molecule studies (Das et al., 2019). Most of the interactions with PIP2 were suggested to happen at the N-termini of the transporters, which are not part of the experimental structures as they have been removed due to their disordered characteristics. Unlike hSERT and dDAT whose structures represent monomeric forms, LeuT was observed to form a dimer in the crystal lattice. Interestingly, the dimeric interface was recently observed to be accentuated by the presence of cardiolipin in prokaryotic membranes (Gupta et al., 2017). Structural studies of effect of lipids on NSS function and oligomerization would be an interesting prospect to explore in the near future.

## Roles and Dynamics of Cytosolic Domains

In the recent past, multiple regulatory roles and cellular functions were attributed to the N and C terminal domains of neurotransmitter transporters. The hSERT and hDAT N and C termini were modeled and were found to have large stretches of disorder interspersed with α-helices and β-strands (Fenollar-Ferrer et al., 2014; Khelashvili et al., 2015). Multiple cellular factors were observed to interact with one or more SLC6 members leading to the regulation of neurotransmitter transporter levels and transporter activity (Table 1). In addition, the C-terminus of hDAT is known to interact specifically with PICK1, a PDZ domain containing protein whose overexpression alongside DAT enhances the cellular uptake of dopamine into cells due to improved cell membrane localization of DAT (Torres et al., 2001) (Table 1). Besides PICK1, hDAT N-terminus is suggested to interact with PIP2, a widely known signaling lipid, which consequently aids in improving amphetamine induced efflux in hDAT (Hamilton et al., 2014). The N-terminus of hDAT is observed to undergo phosphorylation through the activation of Ca^2+^/calmodulin-dependent protein kinase II (CamKII) through binding to the C-terminus of hDAT. Phosphorylation of CamKII is known to induce amphetamine induced dopamine efflux in the dopamine transporter (Fog et al., 2006). The N-terminus of neurotransmitter transporters such as hDAT, hNET, hSERT and hGAT is known to interact with syntaxin-1A, a molecule that is part of the SNARE complex involved in vesicle exocytosis (Quick, 2006) (Table 1). The serine and threonine residues in the N-terminus of hDAT, hNET and hSERT were observed to get phosphorylated through protein kinase C (PKC) *in vivo*, consequently having effects on efflux properties of the transporter and also control the internalization of the transporter through endocytosis (Vaughan, 2004). Most structural studies performed on NSS members tend to have truncated portions of N and C-termini due to their apparent disorder thus limiting the structural understanding of their interacting partners (Penmatsa et al., 2013; Coleman et al., 2016). However, their ability to interact with numerous intracellular factors influence neurotransmitter transport and have consequences for neuropsychiatric conditions (Melikian, 2004). It is, however, not very apparent from a structural perspective as to how these binding partners interact with the termini.

**Table 1:** Intracellular interacting partners of neurotransmitter transporters.

**S. no**	**Transporter**	**Terminus**	**Interacting partner**	**Effect**
1	Dopamine transporter (DAT)	N-Terminus	Syntaxin-1A (Binda et al., 2008)	Increased efflux
			PIP2 (Hamilton et al., 2014)	Increased efflux
		C-terminus	PICK-1 (Bjerggaard et al., 2004)	Improved surface expression
			α-synuclein (Lee et al., 2001)	Reduced uptake
			Hic-5 (Carneiro et al., 2002)	Reduced surface expression
			CamKII (Fog et al., 2006)	Phosphorylation of N-terminus.
		ILs	DJ-1 (Luk et al., 2015)	Increased influx
2	Serotonin transporter (SERT)	N-terminus	Syntaxin-1A (Quick, 2002)	Reduced uptake
			Nitric oxide synthase (Chanrion et al., 2007)	Uptake inhibition
			SCAMP-2 (Muller et al., 2006)	Reduced surface expression
		C-terminus	MacMARCKS (Jess et al., 2002)	Reduced transport
			Hic-5 (Carneiro and Blakely, 2006)	Reduced surface expression
3	GABA transporter (GAT)	N-terminus	Syntaxin-1A (Fan et al., 2006)	Reduced transport rate
		C-terminus	Pals-1 (McHugh et al., 2004)	Improved stability Better transport rate
			Ezrin (Imoukhuede et al., 2009)	GAT1-Actin tether
4	Glycine transporter (GlyT)	N-terminus	Syntaxin-1A (Geerlings et al., 2000)	Reduced rates

## Conclusions

**Figure 10:**
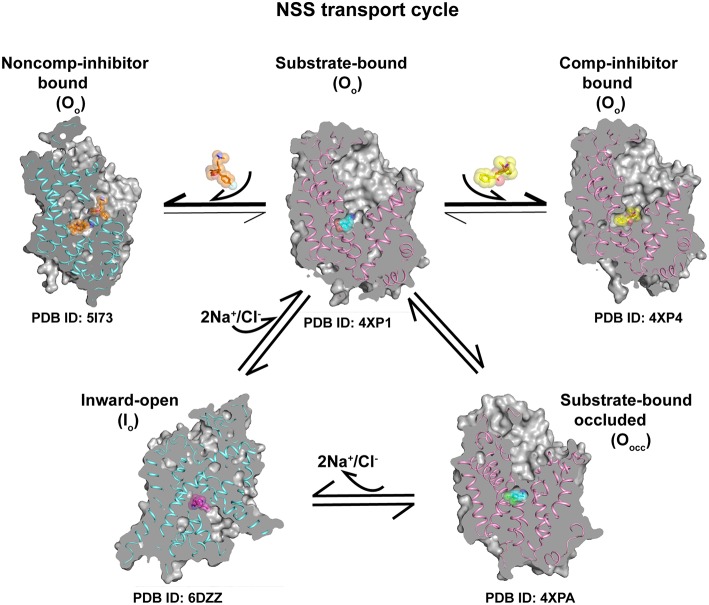
Transport cycle involving a combination of different conformations of the X-ray/cryoEM structures of eukaryotic NSS members dDAT (pink ribbon) and hSERT (blue ribbon) indicating the progression of neurotransmitter transport cycle and conformations stabilized by binding of competitive inhibitors at the primary site and non-competitive inhibition at the allosteric site. Substrate bound O_o_ state can undergo conformational shift to form O_occ_ conformation followed by Io state to establish the transport cycle. Competitive and non-competitive inhibitors lock the transporter and prevent the formation of other conformational states.

The studies in the recent past based on the dDAT and hSERT structures have yielded rich information on the transport and inhibitory mechanisms involved in neurotransmitter transport. With the rather remarkable insight into the inward-open state using cryoEM structure of hSERT, it is now possible to reconstruct the transport cycle of eukaryotic NSS members (Figure 10). The conservation of the features that drive transport in the LeuT, dDAT, and hSERT is extensive with the transport occurring through an asymmetric opening and closure of gating helices TM1 and 6 resulting in the alternate-access of neurotransmitter transport. This “asymmetric rocking-bundle” in all likelihood, constitutes the primary mechanism of transport for the other plasma membrane neurotransmitter transporters, whose structures are yet to be elucidated. Newer roles of the N and C-terminal domains of neurotransmitter transporters are being explored. Recent studies have also indicated evidence for dimerization of eukaryotic NSS transporters particularly through lipid-mediated interactions. Future forays into the structure-function studies of the NSS members would involve extensive computational and biophysical analyses of the cytosolic domains and their roles in regulating transport function.

## Author Contributions

DJ and SP contributed to preparing figures and editing the manuscript. AP wrote the manuscript with inputs from DJ, AM, and SP.

### Conflict of Interest Statement

The authors declare that the research was conducted in the absence of any commercial or financial relationships that could be construed as a potential conflict of interest.

## Abbreviations

ADHD: Attention deficit hyperactivity disorder
CamKII: Calmodulin-dependent protein kinase II
CHS: Cholesterylhemisuccinate
DCP: Dichlorophenylethylamine
dDAT: Drosophila dopamine transporter
EL: Extracellular loop
HDX: Hydrogen deuterium exchange
hGAT: Human GABA transporter
hNET: Human norepinephrine transporter
hSERT: Human serotonin transporter
IL: Intracellular loop
I_o_: Inward-open conformation
I_occ_: Inward-occluded conformation
LeuT: Leucine transporter
NSS: Neurotransmitter sodium symporters
O_o_: Outward-open conformation
O_occ_: Outwardoccluded conformation
PICK1: Protein interacting with C-kinase 1
PIP2, Phosphatidyl inositol 4: 5 bisphosphate
PKC: Protein kinase C
SLC6: Solute carrier 6
SNRIs: Serotonin-norepinephrine reuptake inhibitors
TM: Transmembrane
Tyt1: Tyrosine transporter.
